# Optical pumping and readout of bismuth hyperfine states in silicon for atomic clock applications

**DOI:** 10.1038/srep10493

**Published:** 2015-05-20

**Authors:** K. Saeedi, M. Szech, P. Dluhy, J.Z. Salvail, K.J. Morse, H. Riemann, N.V. Abrosimov, N. Nötzel, K.L. Litvinenko, B.N. Murdin, M.L.W. Thewalt

**Affiliations:** 1 Department of Physics, Simon Fraser University, Burnaby, BC V5A 1S6, Canada; 2 Walter Schottky Institute, Technische Universität München, Am Coulombwall 4, 85748 Garching, Germany; 3 Leibniz-Institut für Kristallzüchtung, 12489 Berlin, Germany; 4 Advanced Technology Institute and SEPNet, University of Surrey, Guildford, Surrey, GU2 7XH, UK

## Abstract

The push for a semiconductor-based quantum information technology has renewed interest in the spin states and optical transitions of shallow donors in silicon, including the donor bound exciton transitions in the near-infrared and the Rydberg, or hydrogenic, transitions in the mid-infrared. The deepest group V donor in silicon, bismuth, has a large zero-field ground state hyperfine splitting, comparable to that of rubidium, upon which the now-ubiquitous rubidium atomic clock time standard is based. Here we show that the ground state hyperfine populations of bismuth can be read out using the mid-infrared Rydberg transitions, analogous to the optical readout of the rubidium ground state populations upon which rubidium clock technology is based. We further use these transitions to demonstrate strong population pumping by resonant excitation of the bound exciton transitions, suggesting several possible approaches to a solid-state atomic clock using bismuth in silicon, or eventually in enriched ^28^Si.

A silicon crystal provides an extremely clean environment within which electrons may orbit impurity atoms, analogous to isolated atoms held in vacuum traps^1^,^2^,^3^,^4^. The hyperfine splittings of alkali atoms in vacuum are widely used as microwave frequency standards^5^, while the push for semiconductor-based quantum information technologies has resulted in dramatic improvements of the coherence times for hyperfine states of shallow donors in silicon^3^,^6^. Magnetic resonance detection in alkali atoms is enabled by selective pumping of the D-lines^7^, but the hyperfine splittings of the equivalent transitions to the odd-parity excited states of shallow donors in semiconductors have so far eluded detection. Here we demonstrate well-resolved hyperfine splittings of these transitions in natural silicon doped with bismuth, and use these transitions to observe strong, controllable polarization of the hyperfine states by pumping the donor bound exciton transitions. Our results open the possibility of sub-ns pumping, and bring solid-state frequency standards and masers based on these donor transitions within reach.

The last fifty years have been a period of outstanding developments in the field of atomic frequency standards, reaching frequency stabilities in the 10^−16^ range in hydrogen masers and accuracies better than 10^−15^ in cesium fountains^5^, ytterbium lasers^8^ and even 10^−18^ in a strontium lattice^9^. In many implementations, hydrogen or alkali-metal atoms with a single valence electron are used, and the resonance transition between the two hyperfine levels of the ^2^S_1/2_ ground-state serves as the frequency reference. The splitting, which is in the microwave range (1–10 GHz), is produced by the interaction between the electron spin and the nuclear spin. The splitting is much smaller than *k*_*B*_*T* (except at mK temperature), and a common characteristic of the preparation to enhance the detected magnetic resonance signal in those implementations is the use of state selection by means of optical pumping^10^. The simplest “intensity pumping” scheme involving a third, higher energy state, uses repeated cycles of selective excitation followed by random relaxation to drive population away from the component being pumped. In more sophisticated variants the pumping is a coherent sum of two fields in a classic “Lambda scheme”. The oft used pumping transitions are the D-lines, i.e. ^2^S_1/2_–^2^P_1/2_ or ^2^S_1/2_–^2^P_3/2_ (D_1_ or D_2_ respectively). These excited states are separated by the fine structure introduced by the coupling of the electron spin with its orbital magnetic moment. Each of the excited levels is further split by the hyperfine interaction, though by a smaller amount than the ground state. Diode lasers with appropriate wavelength and line-width are readily available^7^. In this work we investigate the analogous optical pumping in silicon.

Silicon has found uses in metrology for its mechanical properties as the cavity for mHz line-width lasers^11^, and in parallel, electromagnetic control over impurities in silicon is advancing rapidly. Group V donors in silicon have an absorption spectrum that is closely analogous with hydrogenic free atoms because they also have a single electron that is loosely bound to a singly positively charged core. The main difference in silicon is that the Coulomb force is screened by the dielectric constant of the host, and the effective mass of an electron moving in the host is less than the free electron rest mass, reducing the energy scale for the Rydberg transitions to the mid-infrared, and increasing the orbital radius to a few nm^4^. In addition, the anisotropic, multivalley Si conduction band lifts some degeneracies so that e.g. the 2p_0_ state is separated from the 2p_±_.

The 1s(A1) ground state of group V donors in Si is split by the hyperfine interaction between the electron spin (*S*) and the nuclear spin (*I*), with Si:Bi (*I* = 9/2) having the largest zero-field splitting at 30.51 μeV (7.4 GHz)^12^,^13^,^14^. Si:P (*I* = 1/2) has so far exhibited the longest dephasing times of the hyperfine split components, of order 100 sec when the host is isotopically purified ^28^Si (*I* = 0) so as to remove the ^29^Si (*I* = 1/2) present in natural Si^3^,^6^. In those studies the hyperfine components were polarized by pumping with photons much above the donor ionization threshold, but resonant with the donor-bound-exciton (D^0^X) energy. If the neutral donor (D^0^) is thought of as an analog of hydrogen, then an exciton is the semiconductor equivalent of positronium, and D^0^X is analogous with positronium hydride. It is interesting to note that bound excitons were predicted on the basis of this analogy^15^ well before they were first observed^16^. While the transitions of positronium hydride are not a useful tool for studying the energy levels of hydrogen, in semiconductors the D^0^X transitions lie just below the band gap energy, in the near-infrared for Si, and are in many ways more accessible than the Rydberg transitions, which lie in the mid- to far-infrared.

The transitions and energy levels relevant to this study of Si:Bi are shown in Fig. 1. The hyperfine coupling splits the D^0^ ground state into two levels having total spin *F* = *I* + *S* = 5 and *F* = *I*–*S* = 4, separated by 30.51 μeV. The D^0^X ground state has no hyperfine coupling, since the exclusion principle requires the two electrons to form a spin singlet, which cannot couple to the nuclear spin. The D^0^X transitions can be observed at identical energies in emission via photoluminescence (PL), or in absorption, as shown by the spectra on the left hand side of Fig. 1. Due to the indirect band gap of Si, D^0^X absorption is very weak^17^ and the radiative lifetime is very long, with the decay of D^0^X almost completely dominated by nonradiative Auger decay, or autoionization^18^. We therefore measured the D^0^X absorption spectrum indirectly, detecting the carriers generated by the Auger decay of the created D^0^X, using photoconductivity (PC).

Since the hyperfine splitting occurs in the final state for D^0^X emission, the relative intensity of the two components in PL simply reflects the difference in degeneracies of the two hyperfine states (11:9). The relative intensities of the D^0^X hyperfine components in absorption reflects the relative populations in the two D^0^ hyperfine initial states, and if the two populations were in thermal equilibrium at our temperature of 1.5 K, we would have expected the relative intensities to be near unity in the PC spectrum, since the Boltzmann factor nearly cancels the differences in degeneracy. The essentially identical relative intensities seen in the PL and PC spectra indicates that the D^0^ hyperfine states are not in thermal equilibrium under the conditions used to measure the PC spectrum, but are characterized by a higher effective temperature. This likely results from nonresonant photoionization of the D^0^ hyperfine states and recapture of the free electrons, which acts to randomize, or saturate, the two populations.

The Rydberg transitions to the odd-parity orbital excited states (D^0^*) form a series converging to the Bi ionization energy, 70.98 meV^19^. While the hyperfine splitting of the Bi ground state in the D^0^X transitions of Fig. 1 has already been observed^14^, all previous studies of the Rydberg transitions lacked sufficient spectral resolution to have resolved this splitting^19^,^20^. In the Rydberg absorption spectrum shown in Fig. 2, structure due to the Bi hyperfine ground state splitting is seen in all of the transitions. The observed splitting is ΔE = 30.5 μeV, in agreement with previous measurements using spin resonance^21^ and D^0^X spectroscopy^14^. Hyperfine splittings of the D^0^* excited states are negligible due to their p-like orbital structure and much larger spatial extent. The relative intensities of the hyperfine components are very close to the ratios seen in the D^0^X PL and PC spectra in Fig. 1. The improving spectral resolution with decreasing excited final state binding energy seen in Fig. 2 is similar to effects seen^22^ for phosphorus and boron in ^28^Si and must result from an increasing lifetime, and therefore decreasing lifetime broadening, for the higher Rydberg states. The 2p_0_ state for Si:Bi (not shown) is extremely broad due to near-resonance with optical phonons^19^,^20^, which also produces a much smaller but still significant broadening of the 2p_±_ line by reducing the excited state lifetime. The observed series ends near principal quantum number n = 7 due to concentration broadening, i.e. when then wavefunctions are so large that those of neighbouring donors overlap. The line width of the sharpest transitions is only 9 μeV, almost identical to the D^0^X linewidths, indicating that in both cases the linewidths result predominantly from an inhomogeneous broadening of the shared D^0^ ground state energy. This results from the random isotope distribution present in natural Si, which affects primarily the D^0^ ground state, since both the D^0^X and the excited D^0^* wavefunctions have much larger spatial extent and see an isotopic composition nearer to average than does the compact ground state^23^,^24^.

Finally, we investigate the possibility of hyperpolarization by resonant optical pumping. The same tunable single-frequency laser that was used for D^0^X PC spectroscopy was tuned to either the *F* = 4 or *F* = 5 component of the D^0^X transition, and the resulting change in ground state populations was monitored using absorption spectroscopy of the D^0^* Rydberg states. D^0^X are formed only from D^0^ in the hyperfine state to which the laser is tuned, and due to the Auger recombination and random recapture of free electrons onto the ionized donors, population is pumped away from this state and into the other hyperfine state. Under this resonant pumping, all the hyperfine doublets seen in Fig. 2 changed in the same way, with one example detailed in Fig. 3. When the laser was tuned to the *F* = 4 hyperfine component, the Rydberg transitions having the same ground state were reduced and the other components were correspondingly increased. The situation reversed when pumping the *F* = 5 component. The relative population in the two D^0^ hyperfine states was changed by ± 40% as a result of this optical pumping.

We turn now to possible implications and future directions. We have used the D^0^X transitions for polarization, and the D^0^* transitions for readout, simply because we do not at present have a single frequency source at the D^0^* energies. However, the D^0^* energies are very close to standard III-V Quantum Cascade Laser wavelengths (commercially available down to ~80 meV) and well within the range of other solid state laser sources based on difference frequency generation^25^ or air-plasma generation^26^ (techniques with wavelength ranges that overlap). Unlike the D^0^X transitions, the dipole moment matrix element for the D^0^* transitions is very large (〈x〉~0.28 nm for the 2p_0_ transition^2^), and larger than those of free alkali atoms. We would expect a similar hyperpolarization process when resonantly pumping a D^0^* hyperfine component, even though this would not lead to autoionization as for pumping D^0^X, since memory of the original hyperfine state would be lost during the relaxation cascade from the D^0^* state back to the D^0^ ground states. The short lifetime of the D^0^* states, ~200 ps or less^1^,^27^, compares favorably with the ns to 100’s of ns lifetimes of the D^0^X^18^,^28^. While we have used both transitions in this study, an atomic clock based on the Bi ground state splitting could use either the D^0^X or the Rydberg transitions for both state preparation and readout.

While our observed optical polarization is already quite large it should not represent the ultimately achievable limit. As discussed previously, we believe that the linewidths of both the D^0^X and D^0^* optical transitions is limited by inhomogeneous isotope broadening inherent to natural Si, which can be essentially eliminated using highly enriched ^28^Si as has been shown before for the optical transitions associated with the shallower donor phosphorus and the shallow acceptor boron^24^. The achievable zero field Bi polarization in natural Si is limited by the mismatch between the very narrow (sub MHz) laser linewidth and the ~2 GHz inhomogeneously broadened D^0^X absorption linewidth. Note that the inhomogeneous broadening of these optical transitions results primarily from the mass differences between the Si isotopes, and not from the nonzero nuclear spin of ^29^Si^24^. Besides making both the D^0^X and D^0^* optical transitions much sharper, using enriched ^28^Si will have the further advantage of greatly decreasing the linewidth of the microwave transitions between the D^0^ 1s(A1) hyperfine components by eliminating the random magnetic fields due to ^29^Si nuclei which limit Bi magnetic resonance linewidths to ~500 kHz in natural Si^13^,^29^.

The density of donors in our sample is far larger than in typical atomic clock/maser applications. The latter is necessarily low due to the requirement for low collision frequency, whereas the impurities here are fixed in space without the need for high vacuum systems or trapping. The disadvantage of the silicon system is that low temperatures must be used to keep the donors neutral and reduce phonon interactions. Given the rapid advances being made in QCLs and closed-cycle coolers, it seems reasonable to speculate that large hyperpolarized populations could be created in a small system, and that solid state masers and frequency standards might be within reach. It is also important to note that the Rydberg transitions used here provide electric-dipole readout of the coupled electron and nuclear spins, which is important for quantum information applications. This can be many orders of magnitude faster than magnetic dipole readout with microwaves. At the same time, the results point the way to use of Rydberg pumping to produce the polarization, which should be several orders of magnitude faster than the presently employed D^0^X pumping, due to the stronger absorption and faster relaxation.

## Materials and Methods

All experiments were carried out with a sample from the same crystal used in the previous PL study^14^. It is dislocation-free float-zone grown natural Si doped with Bi with concentration 2 × 10^14^ cm^−3^ in a wafer 1 mm thick and 10 mm in diameter, cut perpendicular to the [001] crystal growth axis, and etched in HF/HNO_3_ to remove surface damage. For the noncontact PC measurements^3^,^6^ the sample was mounted in a strain-free manner between copper foil electrodes in superfluid He at ~1.5 K, and excited with a tunable single-frequency Yb-doped fiber laser. The PL spectrum was obtained with 532 nm above-gap excitation of the sample mounted strain-free in superfluid He at ~1.5 K, using a Fourier transform infrared (FTIR) spectrometer and a liquid nitrogen-cooled Ge p-i-n diode detector, and a spectral resolution of 2.5 μeV (0.02 cm^-1^).

The Rydberg absorption spectrum was obtained using FTIR transmission spectroscopy with resolution 2.5 μeV (0.02 cm^-1^), a black body source, and a liquid helium cooled Si:B photoconductive detector^22^. The sample was mounted strain-free in superfluid He at 1.5 K, and illuminated on one edge with 1047 nm light from a diode pumped Nd:YLF laser, which is just above the band edge of silicon and produces a small population of free electrons and holes that neutralise ionized donor and acceptor impurities, reducing the consequent random local electric fields and thus improving the observed line-widths, especially for the higher excited states. For the optical pumping measurements, the sample was also illuminated with the output of the Yb-doped fiber laser tuned to either the *F* = 4 or *F* = 5 transition of the Bi D^0^X. The data used in this work are available to download at 10.5281/zenodo.16940

## Author Contributions

M.L.W.T. and B.N.M. contributed to the design of the project and wrote the paper, which was reviewed by all authors. K.S., M.S., P.D., J.Z.S., K.L.L., K.J.M., M.L.W.T. and B.N.M. took part in the experiment, while H.R., N.V.A. and N.N. grew the bismuth-doped silicon sample.

## Additional Information

**How to cite this article**: Saeedi, K. *et al*. Optical pumping and readout of bismuth hyperfine states in silicon for atomic clock applications. *Sci. Rep.* **5**, 10493; doi: 10.1038/srep10493 (2015).

## Figures and Tables

**Figure 1:**
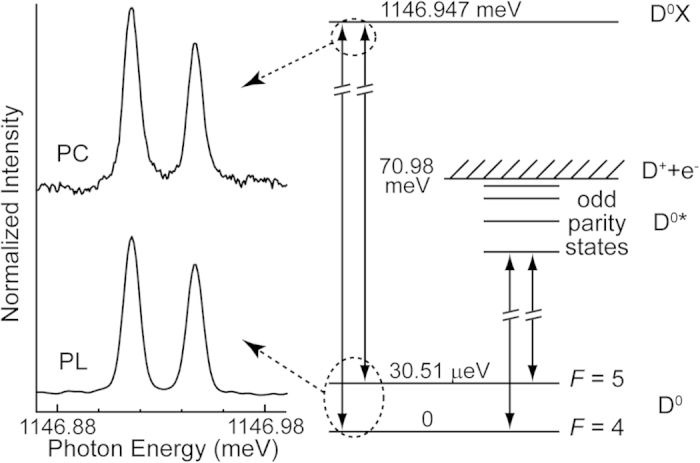
The ground state hyperfine splitting seen in the D^0^X spectrum. The energy levels involved in the Bi donor bound exciton (D^0^X) and Rydberg (D^0^*) transitions are shown on the right hand side. On the left we show spectra of the Bi D^0^X as observed in absorption, using photoconductivity (PC), and in emission, using photoluminescence (PL)^14^.

**Figure 2:**
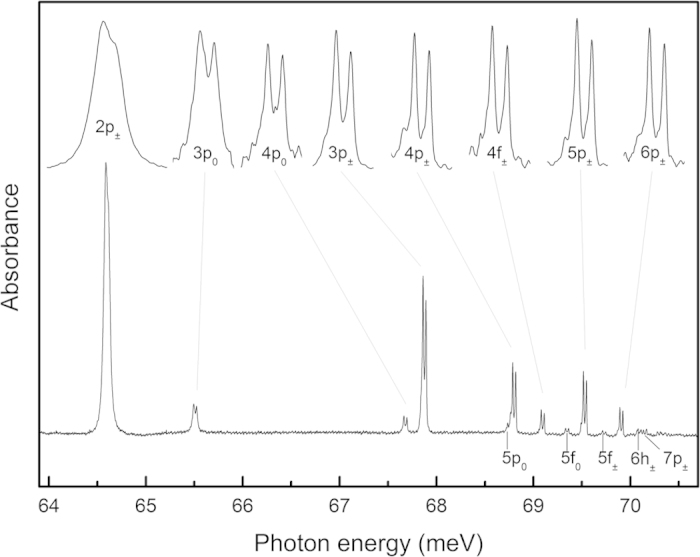
The Rydberg absorption spectrum of Si:Bi at a sample temperature of 1.5 K. Details of major absorption features are shown at the top on an energy scale expanded by a factor of five and arbitrarily shifted, and with normalized intensities.

**Figure 3:**
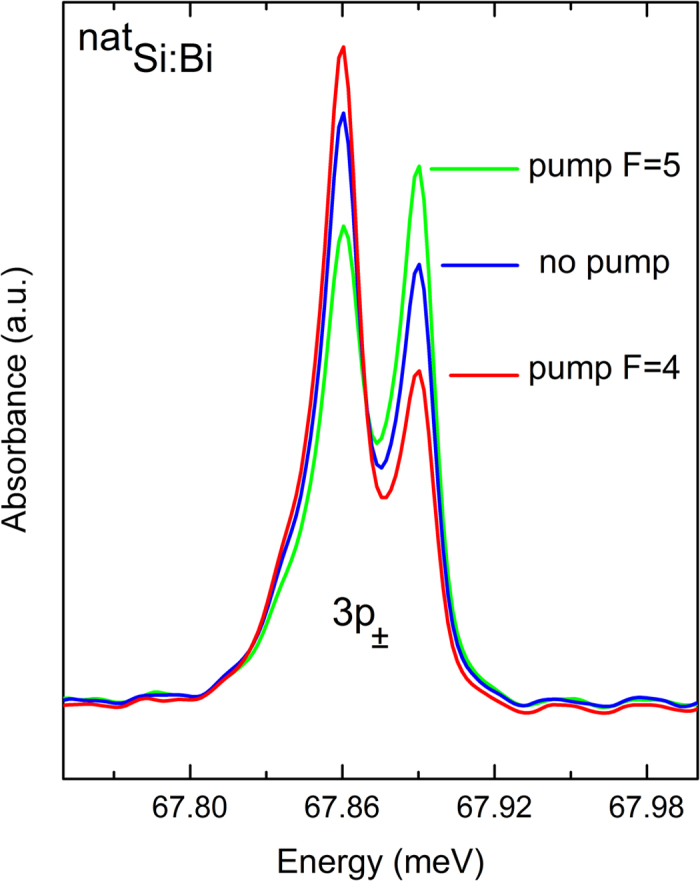
Hyper-polarization of the bismuth D^0^ ground state. Resonant pumping at the D^0^X transition energy produces a change in the relative population of the D^0^ ground state, and consequently a change in the D^0^*absorption spectrum. This example shows the 3p_±_ feature, but all of the absorption transitions reflect the same changes in ground state population. In the absence of D^0^X pumping the relative intensities are as seen in Fig. 2.
